# Digital and algorithmic strategies for guideline-directed medical therapy initiation and up-titration in adults with heart failure with reduced ejection fraction: a systematic review

**DOI:** 10.1097/MS9.0000000000005672

**Published:** 2026-09-10

**Authors:** Naveen Gautam, Saroj GC, Preeti Kharel, Raj Deep Khatiwada, Bibhusan Basnet

**Affiliations:** a Department of Internal Medicine, Gulmi Durbar Basic Hospital, Gulmi, Nepal; b Department of General Medicine, Kakani Primary Health Care Centre, Kakani, Nuwakot, Nepal; c Department of Internal Medicine, Nepal Medical College and Teaching Hospital, Kathmandu, Nepal; d Department of Internal Medicine, MRS Pranami Hospital, Sunsari, Nepal; e Faculty Instructor, Section of Hospital Medicine, Wake Forest University School of Medicine, Winston-Salem, North Carolina, USA

**Keywords:** digital health, guideline-directed medical therapy, heart failure with reduced ejection fraction, remote monitoring, telemedicine

## Abstract

**Background:**

Guideline-directed medical therapy (GDMT) reduces morbidity and mortality in heart failure with a reduced ejection fraction (HFrEF), yet underuse and suboptimal up-titration remain common. Digital and algorithmic strategies are increasingly being used to address these gaps, but their comparative effectiveness remains uncertain.

**Methods:**

This systematic review followed the PRISMA 2020 guidance and was prospectively registered with PROSPERO (CRD420251268580). PubMed, Embase, and Scopus were searched from inception through 26 December 2025. Randomized and comparative studies enrolling adults with HFrEF were eligible if they evaluated digital, algorithm-based, or telemonitoring strategies designed to facilitate GDMT initiation, titration, or optimization versus usual care.

**Results:**

Six randomized comparative trials involving 3706 participants were included. Interventions comprised provider-facing electronic health record alerts and digitally enabled remote titration or telemonitoring pathways. Five of the six trials showed improvements in medication-optimization outcomes; the single neutral trial was a provider-facing alert intervention that showed no difference in GDMT prescribing at hospital discharge. Remote pathway-based strategies demonstrated the largest effects, including higher titration completion (82.1% vs 53.8% at 6 months; an absolute difference of approximately 28 percentage points) and greater improvements in therapy scores. Alert-based interventions showed mixed findings, including no difference in overall GDMT class prescribing at discharge in one inpatient trial (34% vs 34%). Meta-analysis was not performed because of substantial heterogeneity in interventions, outcomes, and reporting formats.

**Conclusion:**

Digital strategies appear to improve GDMT optimization in HFrEF, particularly when embedded within structured care pathways that enable repeated reassessment and protocol-driven titration. Passive alerts alone appear less effective. Standardized outcome measures and trials evaluating clinical endpoints are needed.

## Introduction

Heart failure with reduced ejection fraction (HFrEF) remains a leading cause of cardiovascular morbidity and mortality, and optimization of guideline-directed medical therapy (GDMT) is central to improving outcomes^[^1–3^]^. Nevertheless, many eligible patients do not receive all recommended medication classes or target doses in routine practice^[^4,5^]^.

HIGHLIGHTS

- This systematic review evaluated digital and algorithmic strategies for optimizing guideline-directed medical therapy in adults with heart failure with a reduced ejection fraction.
- Six randomized comparative trials involving 3706 participants were included.
- Structured remote titration and telemonitoring pathways showed the most consistent improvements in guideline-directed medical therapy (GDMT) optimization.
- Passive electronic health record alerts alone demonstrated mixed and less-consistent effectiveness.
- Future studies should use standardized GDMT outcomes and assess clinical endpoints, such as hospitalization and mortality.

Contemporary registries consistently demonstrate large evidence-to-practice gaps. In the CHAMP-HF registry, more than one-quarter of eligible outpatients were not prescribed an angiotensin-converting enzyme inhibitor, angiotensin receptor blocker, or angiotensin receptor-neprilysin inhibitor; one-third were not prescribed a beta-blocker; and two-thirds were not prescribed a mineralocorticoid receptor antagonist. Only 1% of eligible patients were simultaneously receiving target doses of all 3 foundational classes evaluated in that analysis[4]. Longitudinal CHAMP-HF data further showed that medication initiation or dose escalation over 12 months was uncommon across drug classes. Similar patterns have been observed internationally. In BIOSTAT-CHF, only 22% of patients achieved recommended angiotensin-converting enzyme inhibitor or angiotensin receptor blocker doses, and 12% reached target beta-blocker doses[6]. In REPORT-HF, only 37% of patients at discharge and 34% at 6 months were receiving all three recommended classes, with lower rates in low- and middle-income settings[7].

The gap in GDMT implementation is multifactorial and reflects barriers operating at three distinct levels: the patient, the treatment, and the health system. Patient-related barriers include older age, frailty, comorbidity burden, poor adherence, and financial constraints. Treatment-related issues, including intolerance and adverse effects, can further limit optimization[8]. System-level barriers, including therapeutic inertia, fragmented follow-up, repeated laboratory and medication-adjustment requirements, and workflow or reimbursement limitations, also contribute to persistent underuse^[^8,9^]^. These challenges may be amplified by geography; recent data suggest that patients in rural settings receive optimal GDMT less often than urban counterparts and experience worse outcomes[10].

Digital and algorithmic strategies have emerged to address these barriers. Provider-facing tools include electronic health record alerts and clinical decision support, whereas patient-facing or hybrid models include telemonitoring, remote titration platforms, mobile applications, digital consult workflows, and virtual care teams^[^11–14^]^. Early studies have shown mixed results. Some pathway-based interventions have reported faster titration and improved optimization scores^[^12–14^]^, whereas alert-only strategies and dashboard-based quality-improvement approaches have had neutral or more modest effects in some settings^[^11,15–18^]^. Because this literature spans different intervention mechanisms, clinical settings, outcome constructs, and follow-up intervals, the comparative effectiveness of digital strategies for GDMT optimization remains unclear. We therefore conducted a systematic review to evaluate the effectiveness of digital and algorithmic strategies, compared with usual care, for improving GDMT initiation, up-titration, and overall optimization in adults with HFrEF.

## Methods

### Protocol and reporting standards

This systematic review was prospectively registered in PROSPERO (CRD420251268580)[19]. The protocol prespecified quantitative synthesis when at least two studies reported the same outcome using sufficiently comparable methodology, and allowed for narrative synthesis when pooling was not appropriate because of heterogeneity or insufficient data. The review was structured in accordance with the Preferred Reporting Items for Systematic Reviews and Meta-Analyses (PRISMA) 2020 framework[20].

### Eligibility criteria

Studies were eligible if they enrolled adults aged 18 years or older with HFrEF, defined as a left ventricular ejection fraction of 40% or less. Eligible interventions were digital tools or algorithms designed to facilitate initiation, up-titration, or optimization of GDMT. These included provider-facing tools such as electronic health record alerts, best practice alerts, and clinical decision support systems, as well as patient-facing tools such as mobile applications, telemonitoring systems, and remote management platforms that applied software logic to analyze patient data and generate medication prompts or recommendations. Usual care without the specific digital, automated, or algorithmic intervention under study served as the comparator. The protocol allowed randomized controlled trials and nonrandomized comparative studies, including prospective and retrospective cohorts and quasi-experimental studies; however, all studies ultimately meeting the inclusion criteria were randomized comparative trials. Case reports, case series, editorials, commentaries, systematic reviews, meta-analyses, conference abstracts without extractable data, and single-arm studies without a comparator were excluded. Studies in which the digital or algorithmic tool was directed at diagnosis, prognosis, risk prediction, or image analysis rather than at medication initiation, up-titration, or optimization were also outside the scope of the review and were excluded at the search stage using a title-level NOT filter.

### Information sources and search strategy

PubMed, Embase, and Scopus were searched from inception through 26 December 2025. In addition to published reports, the protocol permitted the supplementary identification of relevant unpublished or difficult-to-locate studies through conference proceedings and by contacting authors or domain experts.

The search combined three concept blocks with Boolean operators and applied an explicit exclusion filter. The first block captured the heart failure population, using controlled vocabulary and free-text terms including the MeSH and Emtree heading “Heart Failure” and the terms “HFrEF,” “reduced ejection fraction,” and “systolic heart failure.” The second block captured the digital or algorithmic intervention, using terms and controlled vocabulary such as “clinical decision support” (and “CDSS”), “algorithm*,” “artificial intelligence,” “automated” and “computer-assisted,” “electronic alert,” “best practice alert” (and “BPA”), “nudge,” “telemedicine,” “telemonitoring,” “remote monitoring,” “remote management,” “digital intervention,” “mHealth,” “mobile app,” and “virtual visit.” The third block captured medication initiation and optimization, using terms such as “titration,” “up-titration” (and “uptitration”), “initiation,” “optimization” (and “optimization”), “target dose” and “optimal dose,” “medication management,” “prescribing,” “GDMT,” and “guideline-directed.” Within each block, terms were combined with OR, and the three blocks were combined with AND. To improve specificity, a NOT clause excluded records whose titles indicated a primarily diagnostic, prognostic, risk-prediction, or imaging focus (for example, “diagnosis,” “prognosis” or “prognostic,” “risk prediction,” and “image analysis,” “segmentation,” or “image”), which is outside the scope of this review. Database-specific syntax was applied for each platform, including MeSH headings with appropriate explosion and the [tiab] and [ti] field tags in PubMed, Emtree headings with the tiab and ti field tags in Embase, and adaptation of field tags and truncation symbols for Scopus. The full, line-by-line search strategy for each database is provided in the Supplemental Digital Content Appendix, available at: https://links.lww.com/MS9/B378 No restrictions were placed on language, publication status, or geographic setting. Reference lists of included studies and relevant reviews were also screened to identify additional eligible reports.

### Study selection

Two reviewers independently screened titles and abstracts, followed by full-text assessment of potentially eligible reports. Disagreements were resolved by discussion and, when needed, adjudication by a third reviewer.

### Data extraction

Two reviewers independently extracted data using a structured form. Extracted variables included study design, setting, sample size, intervention characteristics, comparator details, primary and secondary outcomes, follow-up duration, and outcome reporting format. When relevant information was unclear or unavailable in the published report, the protocol permitted contacting study investigators.

### Risk of bias assessment

Risk of bias was assessed with the RoB 2 tool across the following domains: bias arising from the randomization process, deviations from intended interventions, missing outcome data, outcome measurement, and selection of the reported result (Fig. 1)[21]. Two reviewers independently rated each domain, and an overall risk-of-bias judgment of “low risk,” “some concerns,” or “high risk” was derived for every study using the RoB 2 algorithm; disagreements were resolved by discussion and, when necessary, adjudication by a third reviewer. Because all included studies were randomized comparative trials, no nonrandomized appraisal tool was required. The risk-of-bias judgments were used to qualify the certainty of the narrative synthesis rather than to weight a quantitative pooled estimate: greater interpretive weight was given to outcomes from domains rated at low risk, and the implications of the recurring “some concerns” rating for deviations from intended interventions are considered explicitly when interpreting the findings (see the Discussion). No study was excluded on the basis of its risk-of-bias rating, in keeping with the prespecified protocol.

**Figure 1.**
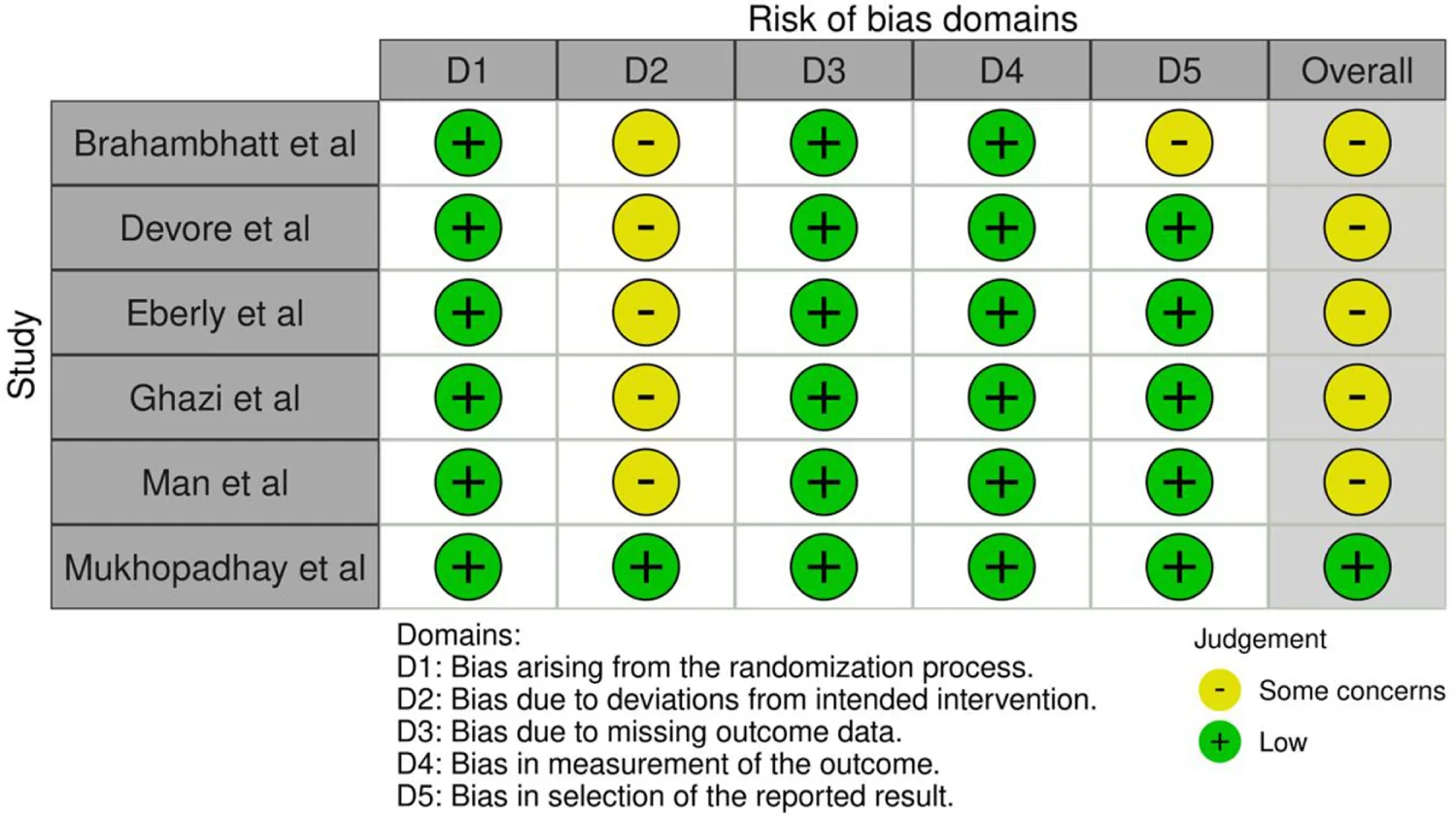
Risk-of-bias summary using RoB 2. All six studies were judged at low risk for bias arising from the randomization process, missing outcome data, and measurement of the outcome. All six studies had some concerns regarding deviations from the intended interventions. Selection of the reported result was judged as some concerns for Brahmbhatt *et al* and as low risk for the remaining studies.

### Synthesis strategy and rationale for narrative synthesis

A meta-analysis was planned only if at least two studies reported the same outcome using sufficiently comparable methodology. The protocol prespecified risk ratios or odds ratios for dichotomous outcomes, mean difference or standardized mean difference for continuous outcomes, and a random-effects model for pooled analyses. Quantitative synthesis was not undertaken because the included studies differed materially in intervention mechanisms, primary outcome definitions, analytic scales, follow-up intervals, and reporting formats. Outcomes included titration completion, changes in composite therapy scores, increases in filled or prescribed GDMT classes, and class-specific prescribing (Table 1). Reporting formats varied across exact event counts, percentages without fully extractable counts, mean ± SD, median (IQR), and adjusted comparative estimates. One study used a stepped-wedge cluster-randomized design and another used a 3-arm cluster-randomized design, further limiting comparability. Accordingly, the evidence was synthesized narratively and summarized in structured tables.

**Table 1:** Primary optimization outcomes and principal barriers to quantitative pooling.

Study	Outcome type	Time point	Intervention result	Control result	Reporting format	Primary barrier to pooling
Brahmbhatt 2024[22]	Titration completion	6 months	46/56 (82.1%)	23/52 (53.8%)	Exact events/totals	Unique endpoint focused on reaching target or maximally tolerated doses
DeVore 2026[13]	Heart failure therapy score change	90 days	1.72 ± 1.75	0.44 ± 1.18	Mean ± SD	Continuous score not comparable with dichotomous outcomes
Eberly 2024[23]	Increase in GDMT classes filled	30 days	66.2%	13.1%	Percentages plus odds ratios	Exact counts unavailable; stepped-wedge design
Ghazi 2023[11]	Increase in prescribed GDMT classes	Discharge	34% of 502	34% of 510	Percentages plus adjusted RR	Inpatient discharge endpoint differs from outpatient longitudinal follow-up
Man 2024[14]	GDMT score change	12 weeks	1.19 (0.25 to 2.30)	0.08 (0.00 to 1.00)	Median (IQR)	Median/IQR reporting and a different score structure
Mukhopadhyay 2023[24]	New MRA prescription	Study period	Alert 224/755; Message 127/812	75/644	Exact events/totals; 3 arms	Class-specific outcome in a 3-arm cluster trial

EHR, electronic health record; GDMT, guideline-directed medical therapy; HF, heart failure; HFrEF, heart failure with reduced ejection fraction; MRA, mineralocorticoid receptor antagonist.

## Results

### Study selection

A total of 3440 records were identified through database searching, including 500 from PubMed, 2,005 from Embase, and 935 from Scopus. After removal of 1763 duplicate records, 1677 titles and abstracts were screened, and 1660 were excluded. Seventeen reports were sought for retrieval, eight full-text reports were assessed for eligibility, and two were excluded, including one pilot study and one preprint. The pilot study was the internal pilot report of the remote titration platform later evaluated in the full Medly Titrate trial (Artanian *et al*[12]), which was excluded to avoid double-counting participants and findings represented by the definitive trial; the preprint was a non–peer-reviewed version of a report that was either superseded by a peer-reviewed publication or lacked separately extractable comparative outcome data. The excluded full-text reports and reasons for exclusion are listed in the Supplemental Digital Content Appendix, available at: https://links.lww.com/MS9/B378. Six studies were included in the final review (Fig. 2).

**Figure 2.**
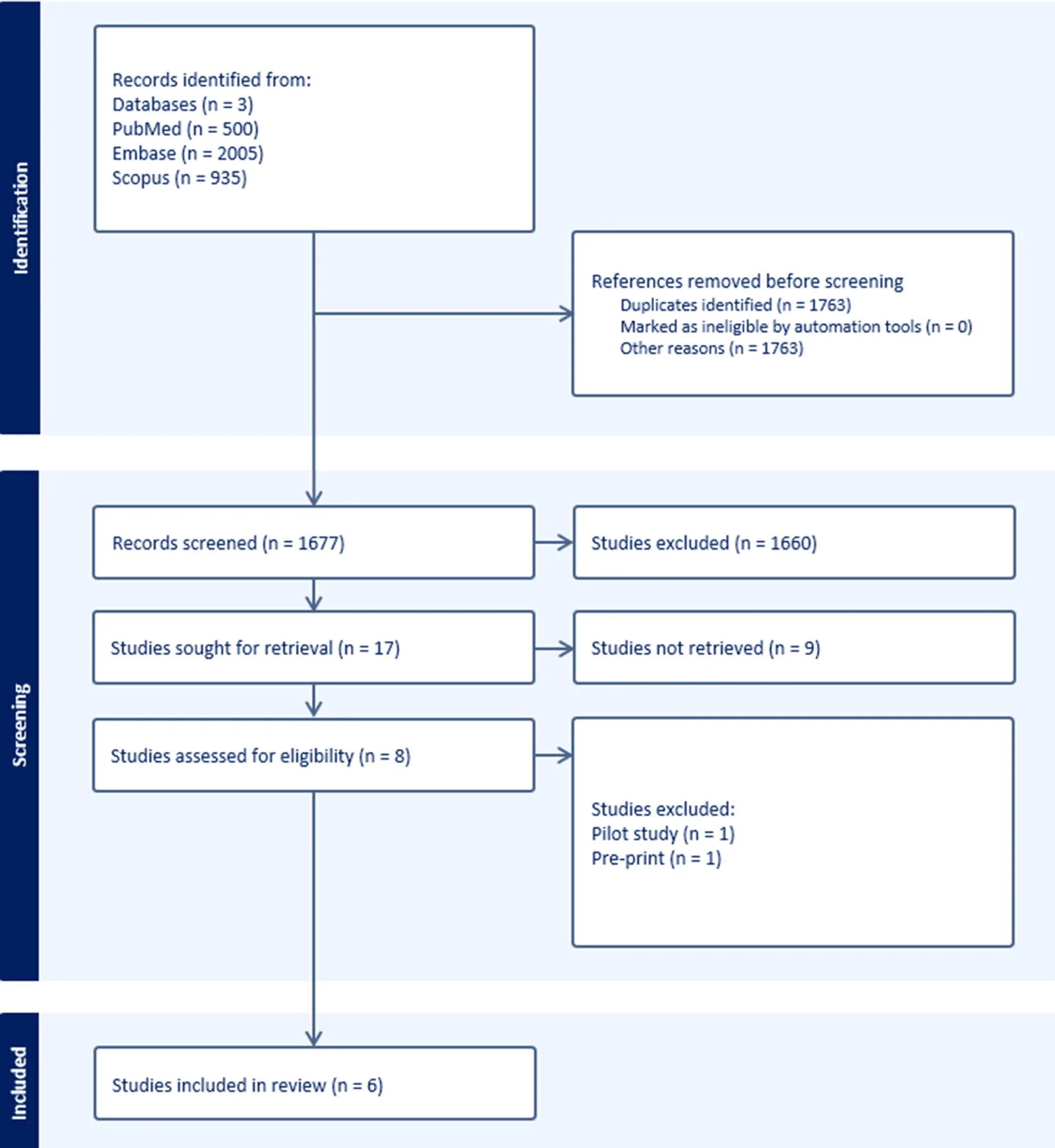
PRISMA 2020 flow diagram of study selection. Database searches identified 3440 records (PubMed: 500; Embase: 2005; Scopus: 935). After removal of 1763 duplicate records, 1677 records were screened; 17 reports were sought for retrieval; 8 full-text reports were assessed for eligibility; and 6 studies were included in the final review.

### Overview of included studies

The six included studies comprised 3706 participants and were published or electronically available between 2023 and 2026 (Table 2)^[^11,13,14,22–24^]^. Three studies were open-label randomized controlled trials: one was a stepped-wedge, cluster-randomized, pragmatic, comparative effectiveness trial; one was a pragmatic multicenter, randomized clinical trial; and one was a 3-arm, pragmatic, cluster-randomized trial. Most studies were conducted in outpatient or home-based settings; one evaluated an inpatient discharge alert strategy during hospitalization for acute heart failure^[^11,13,14,22–24^]^.

**Table 2:** Characteristics of included studies (*N* = 6).

Study	Design	Setting/population	Intervention	Comparator	Primary optimization outcome	Follow-up
Mukhopadhyay 2023 (BETTER CARE-HF)[24]	3-arm pragmatic cluster-randomized trial	Outpatient cardiology practices; adults with HFrEF eligible for MRA initiation; *N* = 2211	EHR-embedded real-time alert during patient encounters, plus a separate asynchronous message arm listing eligible patients	Usual care with silent background alert for timing and eligibility only	Newly prescribed MRA therapy during the study period	During the study period (secondary 30 days)
Ghazi 2023 (PROMPT-AHF)[11]	Pragmatic multicenter randomized clinical trial	Hospitalized adults with acute HF and HFrEF not receiving all four classes of GDMT; *N* = 1012	Personalized provider-facing EHR alert during order entry with tailored GDMT recommendations and order-set links	Usual care with no visible alert	Increase in prescribed GDMT classes at hospital discharge	Index hospitalization (discharge)
Eberly 2024 (Hózhó)[23]	Closed-cohort stepped-wedge cluster-randomized pragmatic comparative effectiveness trial	Rural ambulatory Indian Health Service settings; American Indian adults with HFrEF; *N* = 103	Phone-based telehealth optimization at home with blood pressure cuff monitoring and protocolized treatment review	Usual care during control periods before crossover	Increase in number of GDMT drug classes filled	30 days (repeated across periods)
Brahmbhatt 2024 (Medly Titrate)[22]	Open-label randomized controlled trial	Single-center specialty HF program; adults with HFrEF; *N* = 108	Remote GDMT titration every 2 weeks through the Medly remote patient management platform with rules-based review and nurse-supported care	Usual care titration and management	Completion of GDMT titration to guideline-mandated or maximally tolerated doses	6 months
Man 2024 (ADMINISTER)[14]	Prospective pragmatic multicenter open-label randomized controlled trial	Outpatient clinics; adults with HFrEF; *N* = 150	Digital consult strategy with home data sharing, patient e-learning, and a preconsult digital summary in the EHR; augments a discrete scheduled consultation rather than providing continuous algorithm-driven titration	Usual care HF management	Change in GDMT score (ΔGDMT)	12 weeks
DeVore 2026 (AIM-POWER)[13]	Multicenter open-label randomized clinical trial	Outpatient settings; adults with symptomatic HFrEF not yet optimized on therapy; *N* = 122	BiovitalsHF digital platform using home physiologic data to generate titration recommendations every 2 weeks	Usual care per local practice	Change in heart failure optimal therapy score from baseline	90 days

### Study-level characteristics

The interventions fell into two broad categories. Provider-facing electronic health record notification systems were represented by PROMPT-AHF and BETTER CARE-HF^[^11,24^]^. Active remote titration or digitally enabled management pathways were represented by Medly Titrate, AIM-POWER, Hózhó, and ADMINISTER^[^13,14,22,23^]^. These pathway-based interventions combined patient-generated data, remote assessment, protocolized follow-up, or digital summaries with clinician review to support medication initiation and dose adjustment outside standard episodic visits. These pathway-based strategies were not identical. Medly Titrate and AIM-POWER were continuous remote-titration models in which home physiologic data triggered protocolized or rules-based dose adjustments on a fixed cadence (every 2 weeks) with nurse or clinician oversight, and Hózhó was a telephone-based optimization model with home blood-pressure monitoring tailored to a rural setting. ADMINISTER, by contrast, used a “digital consult” strategy built around a discrete, scheduled clinic consultation: home-collected data, patient e-learning, and a structured preconsultation digital summary were assembled within the electronic health record so that a single planned consultation could be conducted more efficiently and with better information, rather than replacing the visit with continuous algorithm-driven titration. In other words, ADMINISTER digitally augmented an episodic consultation, whereas the other pathways restructured follow-up into a continuous remote process.

In Medly Titrate, completion of GDMT titration at 6 months was higher with the intervention than with usual care [82.1% (46/56) vs. 53.8% (23/52)][22]. In AIM-POWER, the mean change in heart failure optimal therapy score from baseline to 90 days was greater in the intervention group than in usual care (1.72 ± 1.75 vs. 0.44 ± 1.18)[13]. In Hózhó, an increase in the number of GDMT drug classes filled at 30 days occurred in 66.2% of intervention periods versus 13.1% of usual-care periods. In ADMINISTER, the median change in GDMT score over 12 weeks was 1.19 (IQR: 0.25 to 2.30) in the intervention arm and 0.08 (IQR: 0.00 to 1.00) in usual care[14].

Among alert-based strategies, PROMPT-AHF found no difference in the proportion of patients with an increase in prescribed GDMT classes at discharge (34% vs 34%; adjusted risk ratio: 0.95; 95% CI: 0.81 to 1.12)[11]. BETTER CARE-HF demonstrated higher mineralocorticoid receptor antagonist initiation with a real-time alert than with asynchronous messaging or usual care (224/755 vs 127/812 vs 75/644, respectively)[24]. Because BETTER CARE-HF used a 3-arm cluster-randomized design, all three arms are presented above for transparency; the comparison of primary interest for this review was the real-time alert versus usual care, since that contrast most directly isolates the effect of an active provider-facing prompt, whereas the asynchronous messaging arm is reported as an intermediate, lower-intensity alert strategy and was not treated as the principal comparison.

### Primary optimization outcomes

Across the six trials, five reported a favorable effect of the intervention on the primary medication-optimization outcome, whereas one reported a neutral primary result. The strongest signals of benefit came from interventions that embedded digital tools within active titration workflows or structured longitudinal follow-up. In contrast, provider-facing alert strategies showed mixed performance. Taken together, the included studies suggest that digital interventions are more likely to improve GDMT optimization when they function as part of a repeated, protocolized care pathway rather than as passive point-of-care prompts alone.

### Why meta-analysis was not performed

A pooled meta-analysis was not performed because the primary outcomes were not sufficiently comparable for valid aggregation. Completion of titration, change in heart failure optimal therapy score, change in GDMT score, increase in filled drug classes, increase in prescribed drug classes at discharge, and new mineralocorticoid receptor antagonist prescribing are related but distinct constructs. Reporting formats were also incompatible, and time horizons ranged from discharge to 6 months. Heterogeneity extended beyond the outcome definitions to several other dimensions. The healthcare contexts differed substantially, spanning a single-center specialty heart failure program, multicenter outpatient clinics, inpatient acute heart failure care, and rural Indian Health Service settings, each with different baseline prescribing patterns, staffing, and access to specialist follow-up. The interventions themselves were mechanistically distinct, ranging from purely provider-facing electronic health record alerts to nurse-supported remote titration platforms, home physiologic monitoring with algorithm-generated recommendations, and digital consult workflows, such that pooling would have averaged across fundamentally different active ingredients. The enrolled populations also varied in ejection fraction thresholds, baseline therapy, acuity, and demographic composition. Together, these clinical and methodological differences would have produced a pooled estimate that was difficult to interpret and potentially misleading, even if the statistical heterogeneity could be quantified. We additionally considered pooling more consistently reported secondary outcomes such as all-cause hospitalization and mortality; however, these endpoints were reported inconsistently, were generally underpowered in individual trials given their short follow-up, and were defined over differing time windows, so quantitative synthesis of secondary outcomes was likewise not appropriate.

### Risk of bias

Risk of bias was assessed using RoB 2.21. All six studies were judged to be at low risk of bias for the randomization process, missing outcome data, and outcome measurement (Fig. 2). All six had some concerns regarding deviations from intended interventions. Selection of the reported result was judged as low risk in five studies and as some concerns in one study (Brahmbhatt *et al*). Overall, the body of evidence was judged to have some concerns, driven primarily by the open-label and pragmatic nature of the interventions. Deviations from intended interventions were a particular concern in this body of evidence because none of the trials could feasibly blind clinicians or patients to allocation. When a treating clinician knows that a patient is in a digital-optimization arm, that awareness can itself prompt more attentive prescribing, closer follow-up, or earlier dose escalation independent of the digital tool, and because most primary outcomes were clinician-driven prescribing or titration measures, this performance bias could inflate the apparent effect of the intervention. This consideration tempers the certainty of the observed benefits and is weighed in the interpretation below.

*RoB 2 domains* are D1, bias arising from the randomization process; D2, bias due to deviations from intended interventions; D3, bias due to missing outcome data; D4, bias in measurement of the outcome; and D5, bias in selection of the reported result.

The key characteristics of the included studies, including study design, setting, participant population, intervention, comparator, primary optimization outcome, and follow-up duration, are summarized in Table 1.

The primary optimization outcomes, reporting formats, and methodological features that contributed to the decision not to perform a quantitative meta-analysis are summarized in Table 2.

## Discussion

This systematic review synthesized evidence from six randomized comparative trials evaluating digital and algorithmic strategies for GDMT optimization in adults with HFrEF. Across the included studies, pathway-based interventions that incorporated repeated reassessment, remote monitoring, protocolized review, or digitally supported follow-up were associated with more consistent improvements in optimization outcomes than passive provider alerts alone.

The clearest pattern across the evidence base is that digital tools appear most useful when they restructure the cadence of care rather than simply present information. Medly Titrate, AIM-POWER, Hózhó, and ADMINISTER each embedded digital infrastructure within an active process of follow-up and medication adjustment, and each demonstrated improvement in a primary optimization outcome^[^13,14,22,23^]^. This interpretation is supported by STRONG-HF, a non-digital trial showing that high-intensity post-discharge follow-up with rapid titration improves clinical outcomes^[3]^ and by a secondary analysis demonstrating that a higher achieved proportion of optimal dosing is associated with lower short-term mortality[25]. Together, these findings suggest that the active ingredients of successful optimization are repeated reassessment, clear protocols, and accountability for dose escalation.

By contrast, alert-based strategies were less consistent. PROMPT-AHF, an inpatient provider-facing alert trial, did not improve overall discharge optimization despite tailored recommendations and order-set links^[11]^. BETTER CARE-HF improved class-specific prescribing for mineralocorticoid receptor antagonists[24], indicating that alerts may still be useful for discrete, high-yield prescribing actions, particularly when they target underused therapies. Workflow fit likely matters. In the BETTER CARE-HF context, clinician perceptions suggested that real-time alerts integrated more naturally into practice than asynchronous messages[26], and broader implementation evidence indicates that decision-support tools work best when linked to structured follow-through rather than deployed as isolated reminders[27].

These findings have practical implications for health systems seeking to close persistent GDMT gaps. Structured optimization models involving remote monitoring, nurse- or pharmacist-supported titration, and standardized protocols may be more effective than single-encounter alerts^[^28–30^]^. At the same time, the scalability of these interventions deserves careful attention. Most included trials were conducted in academic or integrated health systems with established digital infrastructure, and real-world implementation may be limited by staffing requirements, cost, broadband access, digital literacy, and electronic health record integration^[^9,10,18,31–34^]^. Cost and scalability are central to any adoption decision and warrant emphasis rather than mention in passing. The pathway-based models that performed best were also the most resource-intensive, typically requiring dedicated nursing or pharmacist time, recurring remote contacts every 1–2 weeks, devices for home monitoring, and ongoing technical support and electronic health record integration. None of the included trials reported formal cost-effectiveness or budget-impact analyses, so the value proposition of these programs relative to their staffing and technology costs remains unquantified. The economic case is likely to depend on whether improved prescribing translates into reduced hospitalization, and on whether reimbursement models support remote, team-based titration. Provider-facing alerts, although less consistently effective, are comparatively inexpensive and easy to scale once built into the electronic health record, which may make them an attractive first step or a complement to more intensive pathways in resource-constrained systems.

Equity and usability also merit explicit attention because digital interventions carry a risk of widening existing disparities if they depend on broadband access, smartphone ownership, or digital literacy that are unevenly distributed. Notably, the Hózhó trial, conducted in a rural Indian Health Service population, deliberately used a telephone-based rather than app-based approach and still demonstrated benefit, illustrating that lower-technology designs can extend reach to populations that might otherwise be excluded by more device-dependent platforms. Few of the included studies described patient or community co-design of the intervention, and none reported usability outcomes or differential effectiveness across socioeconomic or demographic subgroups. Engaging patients, caregivers, and frontline clinicians in the design of these tools and reporting their acceptability and accessibility will be important to ensure that digital optimization narrows rather than widens the evidence-to-practice gap.

This review has several strengths. It was prospectively registered, followed PRISMA 2020 guidance, and focused on randomized comparative evidence^[^19,20^]^. It also provides a clinically useful distinction between passive alerts and active digital care pathways. However, several limitations should be acknowledged. The interventions, outcome definitions, reporting formats, and follow-up durations were heterogeneous, precluding meta-analysis and limiting direct comparison across approaches. Most studies were pragmatic and open-label, resulting in some concerns about deviations from intended interventions. Outcomes were primarily process measures rather than hard clinical endpoints, and long-term sustainability remains uncertain. CONNECT-HF, a large post-discharge quality-improvement initiative, did not improve rehospitalization or mortality and showed limited numerical improvement in GDMT over time, underscoring that implementation gains cannot be assumed to translate automatically into patient-level benefit[32]. The short follow-up of the included trials, which ranged from hospital discharge to 6 months, is an important constraint on interpretation. These windows capture whether a digital strategy can accelerate initiation and up-titration but cannot establish whether the gains are durable once active monitoring or protocolized contact ends. Titration intensity often peaks during a structured program, and prescribing may regress toward baseline afterward, so the medium- and long-term trajectory of these effects is unknown; whether early optimization is sustained, and whether it ultimately reduces hospitalization or mortality, will require longer follow-up than any included study provided. Given the small number of trials, their modest sample sizes, the predominantly process-based endpoints, and the “some-concerns” risk-of-bias ratings, these findings are best interpreted as consistent and encouraging signals of feasibility and short-term effectiveness rather than as definitive evidence, and the observed effect sizes should be regarded with appropriate caution pending confirmation in larger trials powered for clinical outcomes.

Future trials should standardize GDMT optimization outcomes, evaluate clinical endpoints in addition to prescribing measures, and compare pathway-based interventions directly with alert-only strategies. Equally important is determining which implementation features are necessary for equitable scale-up in nonacademic and resource-constrained settings. Specifically, priorities for future prospective and randomized research include: adoption of a common core outcome set for GDMT optimization so that results can be pooled across trials; adequately powered randomized trials with follow-up of at least 12 months that assess heart failure hospitalization, mortality, and sustainability of optimization after the intervention ends; head-to-head comparisons that isolate which components of multifaceted pathways (for example, remote monitoring, protocolized titration, or clinician staffing) drive benefit; pragmatic trials embedded in nonacademic, rural, and lower-resource health systems with prespecified analyses of effectiveness across socioeconomic and demographic subgroups; and incorporation of cost-effectiveness, budget-impact, usability, and implementation outcomes alongside clinical endpoints. Addressing these gaps would move the field from demonstrating short-term feasibility toward establishing durable, equitable, and clinically meaningful benefit.

## Conclusion

Digital and algorithmic strategies appear to improve GDMT optimization in HFrEF, particularly when they are embedded in structured, protocol-driven pathways with repeated reassessment. Passive alerts alone are less consistently effective for comprehensive GDMT optimization, though they may still have a role for specific underused therapies, such as mineralocorticoid receptor antagonists, in which a targeted alert improved class-specific prescribing. Passive alerts thus appear better suited to discrete, high-yield prescribing actions than to overall optimization. Standardized outcome definitions and pragmatic trials assessing hospitalization, mortality, and long-term sustainability are needed.

## Data Availability

All data used in this systematic review were obtained from published studies and supplementary materials. Relevant extracted data are included within the article. The full database-specific search strategies and the list of full-text reports excluded with reasons are provided in the Supplemental Digital Content Appendix, available at: https://links.lww.com/MS9/B378.

## Research registration unique identifying number (UIN)

Name of the registry: PROSPERO. Unique identifying number: CRD420251268580. Hyperlink: https://www.crd.york.ac.uk/PROSPERO/view/CRD420251268580.

## References

[1] Greene SJ Butler J Hellkamp AS. Comparative effectiveness of dosing of medical therapy for heart failure: from the CHAMP-HF registry. J Card Fail 2022;28:370–84. 10.1016/j.cardfail.2021.08.023 34793971

[2] Mordi IR Ouwerkerk W Anker SD. Heart failure treatment up-titration and outcome and age: an analysis of BIOSTAT-CHF. Eur J Heart Fail 2021;23:436–44. 10.1002/ejhf.1799 32216000

[3] Mebazaa A Davison B Chioncel O. Safety, tolerability and efficacy of up-titration of guideline-directed medical therapies for acute heart failure (STRONG-HF): a multinational, open-label, randomised trial. Lancet 2022;400:1938–52. 10.1016/S0140-6736(22)02076-1 36356631

[4] Greene SJ Butler J Albert NM. Medical therapy for heart failure with reduced ejection fraction: the CHAMP-HF registry. J Am Coll Cardiol 2018;72:351–66. 10.1016/j.jacc.2018.04.070 30025570

[5] Greene SJ Fonarow GC DeVore AD. Titration of medical therapy for heart failure with reduced ejection fraction. J Am Coll Cardiol 2019;73:2365–83. 10.1016/j.jacc.2019.02.015 PMC7197490 30844480

[6] Ouwerkerk W Voors AA Anker SD. Determinants and clinical outcome of uptitration of ACE inhibitors and beta-blockers in patients with heart failure: a prospective European study. Eur Heart J 2017;38:1883–90. 10.1093/eurheartj/ehx026 28329163

[7] Tromp J Ouwerkerk W Teng THK. Global disparities in prescription of guideline-recommended drugs for heart failure with reduced ejection fraction. Eur Heart J 2022;43:2224–34. 10.1093/eurheartj/ehac103 35393622

[8] Seferović PM Polovina M Adlbrecht C. Navigating between Scylla and Charybdis: challenges and strategies for implementing guideline-directed medical therapy in heart failure with reduced ejection fraction. Eur J Heart Fail 2021;23:1999–2007. 10.1002/ejhf.2378 34755422

[9] Feldman DI Babikian S Feldman T. Leveraging remote patient monitoring to effectively put the heart failure guidelines to practice. J Card Fail 2024;30:1166–69. 10.1016/j.cardfail.2024.04.018 38740175

[10] Mentias A Keshvani N Sumarsono A. Patterns, prognostic implications, and rural-urban disparities in optimal GDMT following HFrEF diagnosis among medicare beneficiaries. JACC Heart Fail 2024;12:1044–55. 10.1016/j.jchf.2023.08.027 37943222

[11] Ghazi L Yamamoto Y Fuery M. Electronic health record alerts for management of heart failure with reduced ejection fraction in hospitalized patients: the PROMPT-AHF trial. Eur Heart J 2023;44:4233–42. 10.1093/eurheartj/ehad512 37650264

[12] Artanian V Ross HJ Rac VE. Impact of remote titration combined with telemonitoring on the optimization of guideline-directed medical therapy for patients with heart failure: internal pilot of a randomized controlled trial. JMIR Cardio 2020;4:e21962. 10.2196/21962 PMC7671843 33141094

[13] DeVore AD Majmudar M Etters L. Digital platform to optimize guideline-directed heart failure therapy: results of the AIM-POWER trial. Circ Heart Fail 2026;19:e013231. 10.1161/CIRCHEARTFAILURE.125.013231 41332416

[14] Man JP Koole MAC Meregalli PG. Digital consults in heart failure care: a randomized controlled trial. Nat Med 2024;30:2907–13. 10.1038/s41591-024-03238-6 PMC11485254 39217271

[15] Verma A Fonarow GC Hsu JJ. DASH-HF study: a pragmatic quality improvement randomized implementation trial for patients with heart failure with reduced ejection fraction. Circ Heart Fail 2023;16:e010278. 10.1161/CIRCHEARTFAILURE.122.010278 PMC10524378 37494051

[16] Scholte NTB Gurgöze MT Aydin D. Telemonitoring for heart failure: a meta-analysis. Eur Heart J 2023;44:2911–26. 10.1093/eurheartj/ehad280 PMC10424885 37216272

[17] Bezerra Giordan L Tong HL Atherton JJ. The use of mobile apps for heart failure self-management: systematic review of experimental and qualitative studies. JMIR Cardio 2022;6:e33839. 10.2196/33839 PMC9015755 35357311

[18] Xu H Granger BB Drake CD. Effectiveness of telemedicine visits in reducing 30-day readmissions among patients with heart failure during the COVID-19 pandemic. J Am Heart Assoc 2022;11:e023935. 10.1161/JAHA.121.023935 PMC9075458 35229656

[19] PROSPERO. International prospective register of systematic reviews. Record CRD420251268580. Accessed 16 March 2026. https://www.crd.york.ac.uk/PROSPERO/view/CRD420251268580.

[20] Page MJ McKenzie JE Bossuyt PM. The PRISMA 2020 statement: an updated guideline for reporting systematic reviews. BMJ 2021;372:n71. 10.1136/bmj.n71 PMC8005924 33782057

[21] Sterne JAC Savovic J Page MJ. RoB 2: a revised tool for assessing risk of bias in randomised trials. BMJ 2019;366:l4898. 10.1136/bmj.l4898 31462531

[22] Brahmbhatt DH Ross HJ O’sullivan M. The effect of using a remote patient management platform in optimizing guideline-directed medical therapy in heart failure patients: a randomized controlled trial. JACC Heart Fail 2024;12:678–90. 10.1016/j.jchf.2024.02.008 38569821

[23] Eberly LA Tennison A Mays D. Telephone-based guideline-directed medical therapy optimization in navajo nation: the Hózhó randomized clinical trial. JAMA Intern Med 2024;184:681–90. 10.1001/jamainternmed.2024.1523 PMC11000136 38583185

[24] Mukhopadhyay A Reynolds HR Phillips LM. Cluster-randomized trial comparing ambulatory decision support tools to improve heart failure care. J Am Coll Cardiol 2023;81:1303–16. 10.1016/j.jacc.2023.02.005 PMC10807493 36882134

[25] Cotter G Deniau B Davison B. Optimization of evidence-based heart failure medications after an acute heart failure admission. JAMA Cardiol 2024;9:114–24. 10.1001/jamacardio.2023.4553 PMC10753435 38150260

[26] Maidman SD Blecker S Reynolds HR. Cardiologist perceptions on automated alerts and messages to improve heart failure care. Am Heart J 2025;279:59–65. 10.1016/j.ahj.2024.10.007 PMC11620927 39423991

[27] Shanbhag D Graham ID Harlos K. Effectiveness of implementation interventions in improving physician adherence to guideline recommendations in heart failure: a systematic review. BMJ Open 2018;8:e017765. 10.1136/bmjopen-2017-017765 PMC5855256 29511005

[28] Dunlay SM Killian JM Roger VL. Guideline-directed medical therapy in newly diagnosed heart failure with reduced ejection fraction in the community. J Card Fail 2022;28:1500–08. 10.1016/j.cardfail.2022.07.047 PMC9588715 35902033

[29] Zheng J Mednick T Heidenreich PA. Pharmacist- and nurse-led medical optimization in heart failure: a systematic review and meta-analysis. J Card Fail 2023;29:1000–13. 10.1016/j.cardfail.2023.03.012 PMC10524094 37004867

[30] Sauer AJ Beon C Cherkur S. Multiregional implementation Initiative’s impact on guideline-based performance measures for patients hospitalized with heart failure: IMPLEMENT-HF. Circ Heart Fail 2025;18:e012547. 10.1161/CIRCHEARTFAILURE.124.012547 PMC12084012 40115978

[31] Tang AB Ziaeian B Butler J. Global impact of optimal implementation of guideline-directed medical therapy in heart failure. JAMA Cardiol 2024;9:1154–58. 10.1001/jamacardio.2024.3023 PMC11447625 39356517

[32] Shoji S Kaltenbach L Granger BB. Guideline-directed medical therapy after hospitalization for acute heart failure: insights from the CONNECT-HF. J Am Heart Assoc 2024;13:e036998. 10.1161/JAHA.124.036998 PMC11935539 39655748

[33] Patolia H Khan MS Fonarow GC. Implementing guideline-directed medical therapy for heart failure: JACC focus seminar 1/3. J Am Coll Cardiol 2023;82:529–43. 10.1016/j.jacc.2023.03.430 37532424

[34] Savarese G Lindberg F Cannata A. How to tackle therapeutic inertia in heart failure with reduced ejection fraction: a scientific statement of the heart failure association of the ESC. Eur J Heart Fail 2024;26:1278–97. 10.1002/ejhf.3295 38778738

